# The temporal dynamics of selective attention are reflected by distractor intrusions

**DOI:** 10.1038/s41598-022-26902-8

**Published:** 2023-01-09

**Authors:** Alon Zivony, Martin Eimer

**Affiliations:** 1 Department of Psychological Sciences, Birkbeck College, University of London, Malet Street, London, WC1E 7HX UK; 2 Department of Psychology, The University of Sheffield, Sheffield, UK

**Keywords:** Psychology, Human behaviour

## Abstract

When observers have to identify an object embedded in a rapid serial visual presentation (RSVP) stream, they often erroneously report the identity of a distractor instead of the target (distractor intrusion). In two experiments, we examined whether these intrusion errors are associated with the speed of attentional engagement. Participants reported the identity of target digits indicated by shape selection cues. To manipulate the speed of engagement, targets appeared either within a single RSVP stream or unpredictably in one of two streams. Objects that followed the selection cue were reported more frequently when engagement was delayed (two streams), whereas the probability of reporting objects preceding the cue was higher when engagement was faster (single stream). These results show that distractor intrusions are closely linked to the allocation of selective attention in time, making the intrusion paradigm a useful tool for research into the temporal dynamics of attention. They also provide new evidence for the idea that attentional selectivity operates within brief periods of perceptual enhancement (attentional episodes), facilitating the processing of all objects within this period, regardless of their status as targets or distractors.

## Introduction

When navigating dynamic environments, our ability to quickly detect and identify important events is critical for our survival. This is evident in tasks such as driving, where noticing an unexpected pedestrian or a car can mean the difference between life and death. Selective attention is essential for such tasks. Selective attention allows us to process and respond to a small portion of the environment by biasing processing in favor of specific areas in space at specific points in time. Therefore, a comprehensive understanding of the function of attention must consider both spatial selectivity and temporal selectivity.

**Figure 1:**
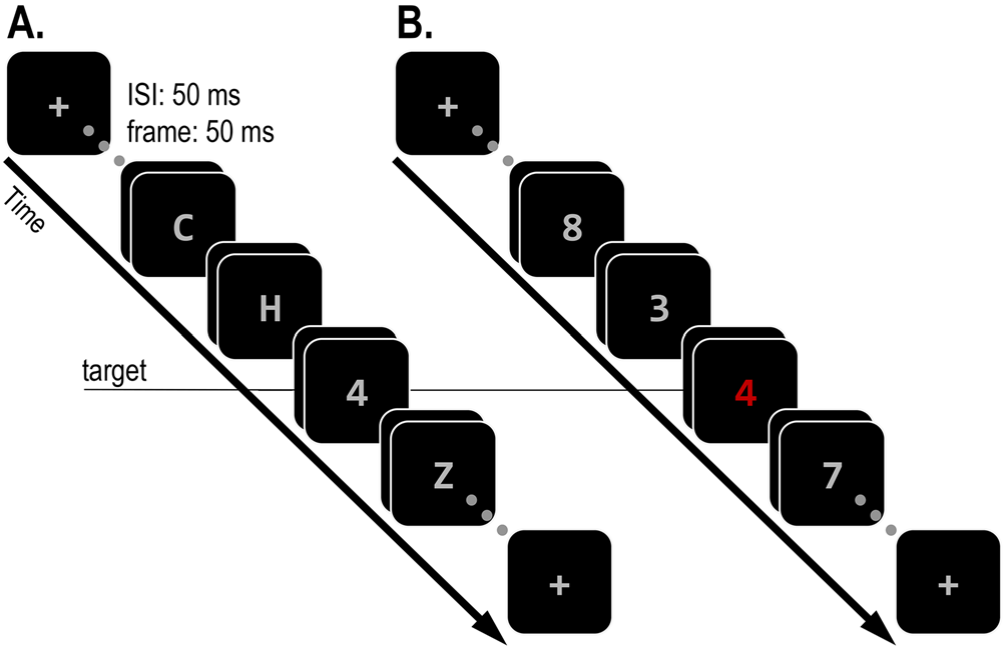
Examples of a traditional RSVP task (**A**) and of a distractor intrusion task (**B**). In both examples, participants have to report the identity of a target digit. In the traditional task, the target is embedded among distractor letters. In the distractor intrusion task, the target is defined by its colour and embedded among distractor digits. Therefore, distractors in the second task can be erroneously reported instead of the target.

In the spatial domain, much research has been conducted to estimate the size and shape of the spatial focus of attention^1–3^. In the temporal domain, many studies have focused on how attending to specific task-relevant points in time can improve performance (attention *to time*; see^4^ for review). In contrast, much less research is concerned with how selective attention unfolds over time (attention *in time*), or in other words, with the temporal dynamics of attention. A possible reason for this is that it is often assumed that the temporal resolution of attention is highly precise^5,6^. This assumption is based on numerous studies using the rapid serial visual presentation (RSVP) task, which have shown that a target can be easily detected even when it is embedded among multiple distractors and appears for less than 100 ms^7^. However, other findings, and in particular, the phenomenon of distractor intrusions^8–12^ (and similar phenomena^13–15^) challenge this assumption. In standard RSVP tasks (e.g., identify a digit among letter distractors), the distractors are not candidates for report (Fig. 1A). In contrast, targets in the distractor intrusion paradigm are embedded among distractors that share the target’s response dimension (e.g., other digits) and are therefore reportable (Fig. 1B). This simple manipulation usually has drastic effects on performance. Accuracy is strongly reduced, as observers often erroneously report the identity of temporally adjacent distractors instead of the target (distractor intrusion errors). Indeed, in a recent study we found that merely changing the distractor that immediately follows the target (post-target distractor) from a non-reportable letter to a reportable digit can reduce accuracy by half^11,12^. This frequency of distractor intrusions errors demonstrates that selective attention is much less temporally precise than is commonly thought.

**Figure 2:**
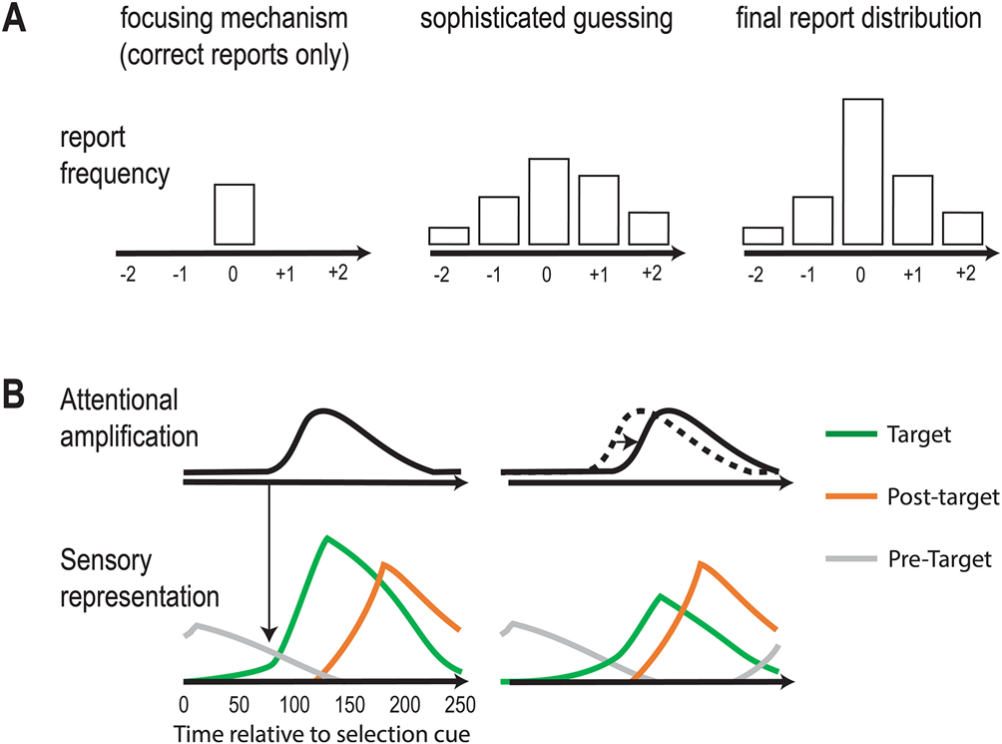
Two accounts of distractor intrusions. (**A**) The selection failure account suggests that two mechanisms underlie response selection in RSVP paradigms: (1) a focusing mechanism that produces correct reports, and (2) a sophisticated mechanism that is engaged only when the focusing mechanism fails. Together, they determine the distribution of distractor intrusion reports across trials. (**B**) According to the attentional episode account, target detection result in attentional amplification that enhances the sensory representations of several successively presented objects in the same location. Since strongly activated representations are more likely to be reported, this can result in distractor intrusions, especially when the onset of the attentional episode is delayed.

Distractor intrusions can in principle help to track the allocation of selective attention in time. However, this will only be possible if these intrusions are directly linked to the temporal distribution of attention. The purpose of the current study is to provide new evidence that this is indeed the case. This is important because one prominent account of distractor intrusions^8^ provides an alternative interpretation. According to this account, distractor intrusions occur first and foremost because attention sometimes fails to select the target. The main factor that influences the success of this focusing mechanisms is the speed in which the target-defining feature is processed. When attentional focusing fails, reports rely on a secondary sophisticated guessing mechanism, where a response is selected from a sampling distribution of available response features (Fig. 2A). This sampling distribution is determined by (*i*) the strength of the objects’ perceptual trace, which is often skewed in favor of objects that follow the target, and (*ii*) the attentional weights, which are symmetrically distributed around the target, based on the temporal proximity of other objects. Since this account postulates that intrusions can emerge only when selection fails, we refer to this proposal as the *selection failure* account.

In contrast to Botella et al., other models assume that variability in the temporal distribution of attention is the critical factor in determining which item will be reported on a given trial^10,12,16,17^. These models suggest that distractor intrusions occur because attentional enhancement triggered by the target continues for a period of time (an attentional episode) and enhances temporally adjacent distractors (Fig. 2B). Responses are made based on the strength of the available sensory representations^10^. Depending on the timing of an attentional episode, a distractor’s response feature may be strongly co-activated at the same time as the target’s selection feature. In this case, the distractor might be encoded either instead of the target or alongside it^11^. If both are encoded, the distractor might be encoded earlier (prior entry^18^) or both items might be encoded as an integrated object, thus eliminating any temporal information (temporal integration^13–15^). The common denominator in all these cases is that, due to the enhancement received by the distractor, this item is sometimes reported instead of the target. Therefore, we shall refer to this view as the *attentional episode* account.

The two accounts have very different implications for research into temporal selectivity. According to the selection failure account, distractor intrusions provide insights about factors determining successful target selection, but cannot teach us about the temporal distribution of attention, which is assumed to be fixed around the position of the target (unlike perceptual traces, which are often stronger for post-target objects). In contrast, according to the attentional episode account, the pattern of correct responses and distractor intrusion errors directly reflects the distribution of attentional selectivity over time. If this account is correct, distractor intrusions can provide us a powerful tool to study the temporal dynamics of attention. Resolving between the two accounts is therefore critical for the application of distractor intrusions in future attention research.

A key factor that differentiates between the two accounts is whether correct reports and distractor intrusions are produced by qualitatively different mechanisms, or are the result of a quantitative difference in the operation of a single mechanism. According to the selection failure account, correct reports can stem from either successful focusing or sophisticated guessing, whereas distractor intrusions always result from sophisticated guessing when focusing fails. In contrast, the alternative attentional episode account postulates no such qualitative difference. Instead, correct reports and intrusions both reflect the relative amount of attentional enhancement received by successive objects, regardless of their objective status as a target or distractor.

We^11,12^ previously provided initial evidence for this hypothesis by measuring electrophysiological markers of attentional engagement (the N2pc component^19–21^) on trials with correct reports versus post-target intrusions. Trials with post-target intrusions were associated with a delay in the onset latency of the N2pc relative to trials with correct reports. Since attentional engagement reflects the transient attentional response following the detection of the target, this finding suggests that intrusions occur when the post-target distractor receives stronger attentional enhancement than the preceding target. In the present study, our goal was to further differentiate between the selection failure and the attentional episode accounts by examining not only post-target intrusions, but also intrusions by objects that precede the target (pre-target distractors). To manipulate the onset of attentional engagement, we presented targets in either a single RSVP stream or unpredictably in one of two RSVP streams. With a single stream, spatial attention can be focused in advance on the target’s location, and this should therefore result in faster detection of the target^22^ and consequently faster attentional engagement, relative to when the target’s location is uncertain^12,23^. If intrusions are linked to the temporal distribution of attentional enhancement, a manipulation that delays attentional engagement should increase post-target intrusions but *reduce* pre-target intrusions (as they shall benefit even less from attentional enhancement). Recently, Ludwici and Holcombe^9^ used a similar manipulation and found evidence for such a pattern. They concluded that perceptual representations of pre-target items persist for a short amount of time and that these buffered representations may be selected for report when attentional engagement is fast enough to promote their processing.

However, the design of the study by Ludwici and Holcombe’s did not allow an independent assessment of how attentional engagement speed affects pre-target and post-target distractor intrusions, respectively. Each RSVP stream always included both a reportable pre-target and post-target object, but only one item could be reported on a given trial; pre-target and post-target reports were interdependent. Thus, their results only showed that delayed engagement was associated with stronger relative bias towards post-target distractors, but not that faster engagement was linked to an absolute decrease in pre-target distractor intrusions. To demonstrate this, a design is required where these two types of distractors do not compete directly for report. Such a design was employed in Experiment 1, where targets were digits that were indicated by a shape cue (square outline). Targets were either preceded and followed by non-reportable distractors (letters; baseline condition), or appeared together with a reportable distractor (another digit) that either preceded or followed the target. This allowed us to measure the absolute likelihood of pre-target and post-target-intrusions, and to assess how each of these distractors affected target accuracy relative to the baseline condition.

**Figure 3:**
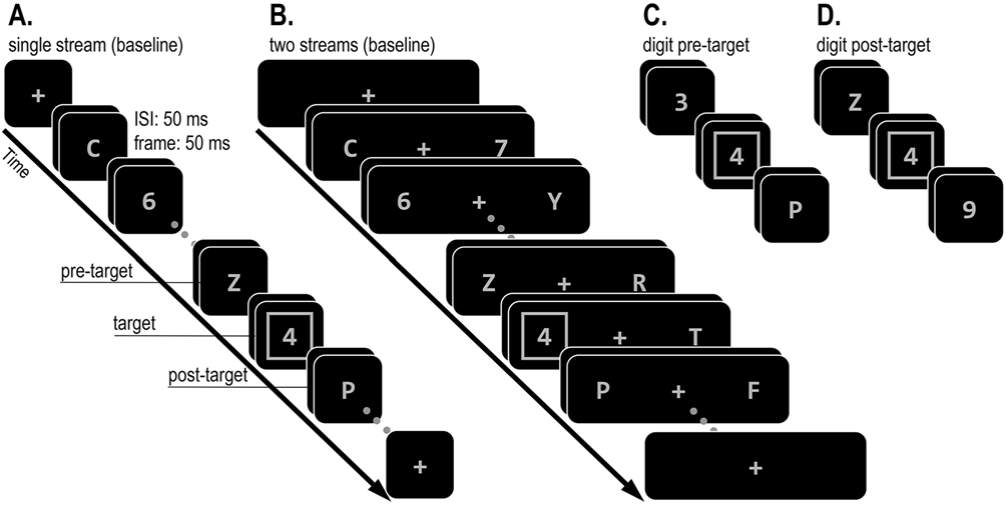
Illustration of the stimulus sequence in Experiment 1. Participants had to report the target digit inside a square. The target digit was embedded among streams of both letters and digits distractors such that only the square (and not its alphanumeric category) could be used to identify the target. The target appeared at positions 5 to 8 within a single (**A**) or two (**B**) streams. The pre-target frame and post-target frames contained only letters (baseline condition; **A**–**B**) on 20% of the trials. On the rest of the trials, either the pre-target frame or the post-target frame contained a digit at the same location as the target (**C**–**D**).

According to the attentional episode account, increasing the number of RSVP streams should have opposite effects on the frequency of pre-target and post-target intrusions: Pre-target intrusions should be more frequent with faster engagement (single-stream condition), whereas post-target intrusions should increase when engagement is delayed (two-stream condition). In contrast, according to the selection failure account, this delay should increase the probability that attentional allocation is not successful, thereby increasing the reliance on the sophisticated guessing mechanism and the overall proportion of intrusions. Importantly, in the absence of competition between the pre-target and post-target distractors (when only one of them is reportable), the guessing mechanism will be forced to select whatever distractor is present. Therefore, the selection failure account predicts a general increase of both types of intrusions (not just of post-target intrusions) with two streams as compared to a single RSVP stream (Fig. 3).

## Experiment 1

### Results

**Figure 4:**
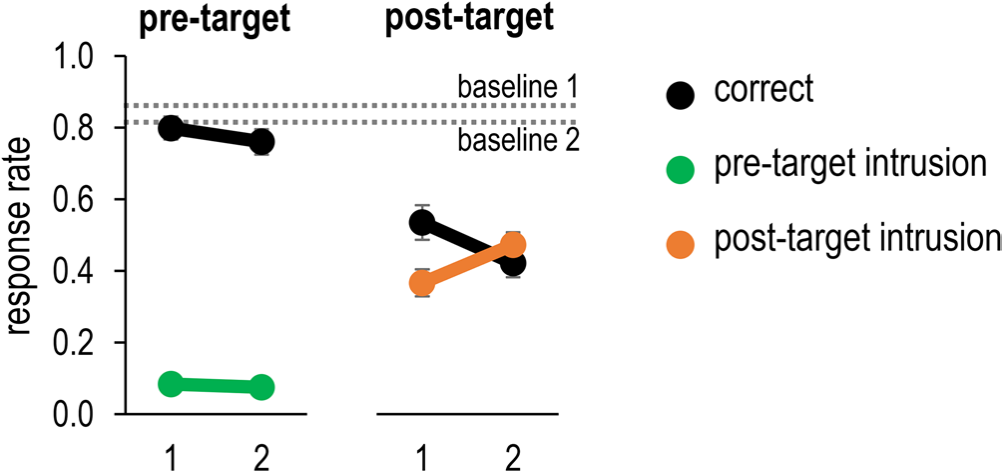
Frequency of correct responses (black lines), pre-target intrusions (green lines) and post-target intrusions (orange lines) in Experiment 1, as a function of the distractor condition (pre-target digit vs. post-target digit) and the number of streams (1 vs. 2). Error bars denote one standard error. The dotted lines represent baseline accuracy (i.e., no pre/post-target digit distractors) based on the number of streams.

Mean accuracy data and intrusions data are presented in Fig. 4.

#### Accuracy

For both the single-stream and the two-streams conditions, accuracy was lower when a potentially intruding distractor (either a pre-target or post-target distractor) appeared alongside the target (all *p*s < 0.01). Accuracy was overall lower in the post-target condition than in the pre-target condition, *F*(1,31) = 91.91, *p* < 0.001, ${\eta }_{p}^{2}$=0.75, indicating a greater interference from the post-target distractor. Moreover, accuracy was lower in the two-streams condition relative to the single-stream condition, *F*(1,31) = 22.96, *p* < 0.001, ${\eta }_{p}^{2}$=0.43. Importantly, the interaction between the two factors was also significant, *F*(1,31) = 7.25, *p* = 0.011, ${\eta }_{p}^{2}$=0.19. This result indicates that the drop in accuracy was much larger in the post-target condition, *t*(31) = 5.68, *p* < 0.001, *d* = 1.01 (*M* = 53.2% vs. *M* = 42.0%) than in the pre-target condition, , *t*(31) = 2.06, p = 0.048, *d* = 0.37 (*M* = 79.6% vs. *M* = 75.2%).

#### Distractor intrusions

The pattern of intrusions was nearly a mirror image of the accuracy data for the post-target condition, but not for the pre-target condition. Intrusion rates were higher in the post-target condition than in the pre-target condition, *F*(1,31) = 92.89, *p* < 0.001, ${\eta }_{p}^{2}$=0.75 (*M* = 42.0% vs. *M* = 7.9%). Importantly, the interaction between the distractor type and the number of streams was significant, *F*(1,31) = 25.42, *p* < 0.001, ${\eta }_{p}^{2}$=0.45. In the post-target condition, intrusion rates were also higher when there were two RSVP streams relative to one RSVP stream (*M* = 36.6% vs. *M* = 47.3%, *p* < 0.001). In the pre-target distractor condition, there was no difference in intrusion rates as function of the number of streams (*M* = 8.4% vs. *M* = 7.5%, *p* = 0.30). Thus, whereas increasing the number of streams always reduced accuracy, it only increased post-target intrusions, but not pre-target intrusions.

One possible reason why pre-target intrusions were not modulated by the number of streams is the presence of floor effects (i.e., too few pre-target intrusions to observe any reliable reduction). To test this possibility, we explored this effect among participants who committed above-average pre-target intrusions, based on a median split of overall pre-target intrusions rate (> 7.5%). Among these participants, we observed a reduction in pre-target intrusions on the two-streams condition relative to the single-stream condition, *t*(12) = 2.86, *p* = 0.014, *d* = 0.79 (*M* = 8.8% vs. *M* = 12.7%).

### Discussion

In Experiment 1, a target digit was embedded in either one or two RSVP streams, which should delay attentional engagement in the two-streams condition. In line with the attentional episode account, target accuracy was reduced and the frequency of post-target intrusions was substantially higher in the two-streams condition relative to the single-stream condition. In contrast, no analogous increase in pre-target intrusions was observed. Instead, the number of pre-target intrusions was generally low and unaffected by the number of the streams.

Thus, the speed of attentional engagement clearly had a different effect on pre-target and post-target intrusions, even though the two types of responses were evaluated on different trials. This finding is incompatible with the selection failure account, which assumes that all intrusions stem from a shared guessing mechanism that kicks-in whenever attention fails to select the target. However, this pattern is also somewhat inconsistent with the attentional episode account. According to this account, a delay in engagement should reduce any sensory enhancement of pre-targets, reducing the chances that this distractor successfully competes with the target at the response selection stage, and thus the frequency of pre-target intrusions. The absence of such a reduction in Experiment 1 may be due to the overall low frequency of pre-target intrusions, as also suggested by the fact that participants who committed more pre-target intrusions did show this effect. However, given that this median-split analysis was post-hoc, it cannot provide sufficiently strong support for a link between the speed of engagement and the frequency of pre-target intrusions. Experiment 2 was conducted to obtain more clear-cut evidence.

## Experiment 2

**Figure 5:**
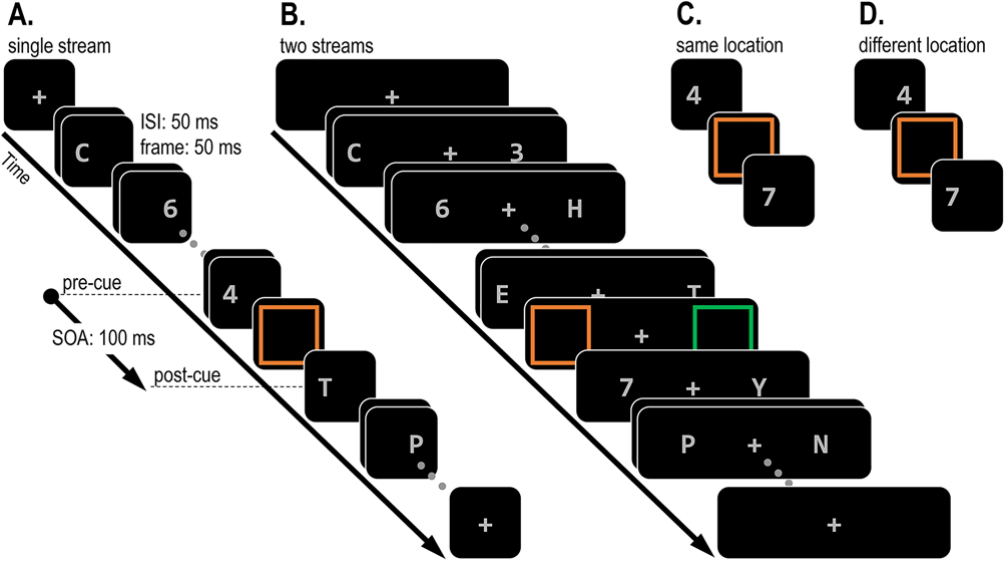
Illustration of the stimulus sequence in Experiment 2. Participants had to report any digit they saw inside a square in a prespecified colour (e.g., orange). The selection cue was embedded in either (**A**) a single stream or (**B**) two streams. In every stream, the item immediately before and immediately after the selection cue appeared in exactly the same location (**C**) or in a different location (**D**). In the pre-cue digit only or post-cue digit only conditions (**A**–**B**; 20% of trials), only one digit appeared either before or after the selection cue. In the other trials (**C**–**D**; 80% of trials), two different digits appeared both before and after the selection cue.

Experiment 2 introduced two major changes that were intended to increase the reporting probability of objects that preceded the selection cue. In Experiment 1, the competition between the pre-target and the target was strongly biased towards the target because it coincided with the selection cue. To eliminate this bias, the selection cue was placed mid-way in the interval between two potential targets (see^10^, for analogous procedure). Note that due to this change, there was no longer a single target. We therefore refer to the objects preceding and following the selection cue as the pre-cue and post-cue digit, respectively. In addition, the processing of the pre-target item in Experiment 1 was likely disrupted due to backward masking by the subsequent target, as both objects appeared at the same location. Therefore, in Experiment 2 objects in each stream could appear in one of two positions, both located within the area covered by the selection cue. Consequently, the pre-cue and post-cue digits could appear in the same location (Fig. 5C) or at different locations (Fig. 5D). We assumed that pre-cue digit reports would be higher in the different-location condition, which should make it possible to observe a stronger impact of the number of streams on these reports.

On 80% of all trials, RSVPs included both pre-cue and post-cue digits. If the speed of engagement was directly linked to the successful encoding of pre-cue versus post-cue digits, the probability of reporting post-cue digits should increase when engagement is slow (two-streams condition). The reverse pattern should be observed for pre-cue digits. These differential effects should be particularly clear in the different-location conditions in the absence of backward masking. On the remaining 20% of trials, only the pre-cue digit or the post-cue digit appeared in the stream, and the other item next to the cue was a letter. These two conditions allowed us to observe the rate of reports for the pre- and post-cue digits in the absence of competition for response selection from another digit. We expected that, analogous to trials where both digits were available for report, pre-cue digits should be less likely to be reported in the two-streams condition, while the reverse should be the case for post-cue digits.

It is more difficult to derive exact predictions for Experiment 2 from the selection failure account for trials with both pre-cue and post-cue digits, given that there was no longer a single target object for selection in most trials. We reasoned that, analogous to Experiment 1, this account should predict that uncertainty about target location should increase the probability of failing to select either of the two possible targets, and thus the activation of the sophisticated guessing mechanism. There should therefore be a similar decrease in both pre-cue and post-cue digit reports in the two-stream relative to the one-stream condition, but no difference between these two types of reports. An equivalent pattern should also be found for trials with only the pre-cue or post-cue digit.

### Results

#### Pre-cue and post-cue digit (Fig. 6A)

**Figure 6:**
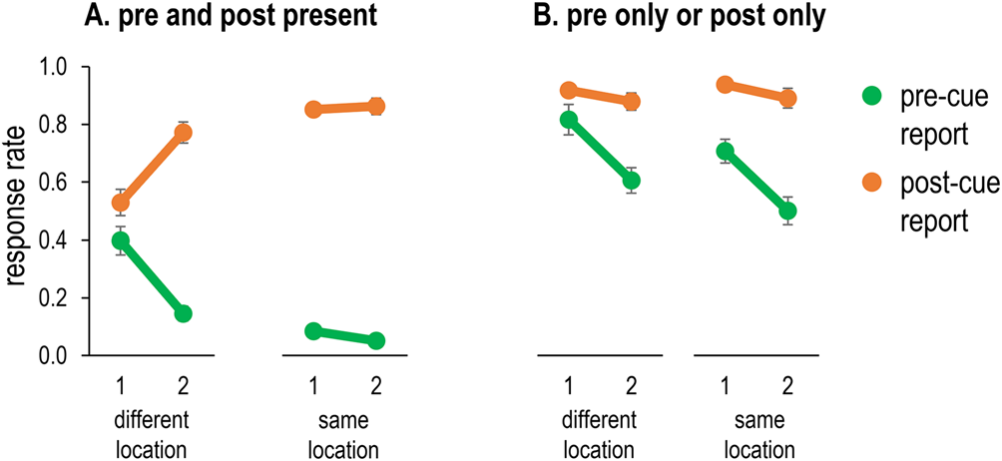
Frequency of pre-cue digit reports (green lines) and post-cue digit reports (orange lines) in Experiment 2, for trials with two digits adjacent to the selection cue (A) and trials where only a pre-cue or post-cue digit was present (B), as a function of the number and the number of streams (1 vs. 2), and the location of the pre- and post-cue items (same vs. different). Error bars denote one standard error.

The three-way interaction between distractor-type, number of streams and pre-cue/post-cue location was significant, *F*(1,15) = 31.72, *p* < 0.001, ${\eta }_{p}^{2}$=0.68. Therefore, we conducted a separate analysis for reports of the pre-cue and the post-cue items. Pre-cue digit reports were more likely in the single-stream condition (*M* = 24.1% vs. *M* = 9.8%, *F*(1,15) = 26.21, *p* < 0.001, ${\eta }_{p}^{2}$=0.64), and in the different location condition (*M* = 27.1% vs. *M* = 6.8%, *F*(1,15) = 49.50, *p* < 0.001, ${\eta }_{p}^{2}$=0.77). These location effects were smaller in the two-streams condition, as indicated by a significant streams × location interaction, *F*(1,15) = 30.14, *p* < 0.001, ${\eta }_{p}^{2}$=0.67. This result likely reflects floor effects on same-location trials (see Fig. 6A). As predicted, the pattern for post-cue reports was the exact opposite of pre-cue reports. Post-cue digit reports were more likely in the two-streams (*M* = 81.7% vs. *M* = 69.0%, *F*(1,15) = 13.68, *p* = 0.002, ${\eta }_{p}^{2}$=0.48), and when the two digits appeared in the same location (*M* = 85.7% vs. *M* = 65.0%, *F*(1,15) = 53.97, *p* < 0.001, ${\eta }_{p}^{2}$=0.78). Moreover, the location effect was smaller in the two-streams condition, *F*(1,15) = 32.51, *p* < 0.001, ${\eta }_{p}^{2}$=0.68. Finally, we note that post-cue reports exceeded pre-cue reports in almost all conditions, except for the single-stream/different-location condition where this comparison did not reach significance, *t*(15) = 1.42, *p* = 0.18, *d* = 0.35. The comparison was clearly significant in the other conditions (all *p*s < 0.001).

#### Pre-cue digit or post-cue digit only (Fig. 6B)

All main effects were significant (*p*s < 0.01), as were the distractor-type × streams and distractor-type × location interactions (*p*s < 0.001). Reports of pre-cue digits were again more frequent when the subsequent item appeared at a different location (*M* = 71.1% vs. *M* = 60.4%, *p* < 0.001), whereas post-cue digit reports were unaffected by the previous item’s location, *F* < 1. In contrast with the previous analysis, both post-cue reports and pre-cue reports were reduced in the two-streams condition relative to the single-stream condition. However, these differences were larger for pre-cue reports, *F*(1,15) = 43.37, *p* < 0.001, ${\eta }_{p}^{2}$=0.74, than for post-cue reports, *F*(1,15) = 7.35, *p* = 0.016, ${\eta }_{p}^{2}$=0.33 ($\overline{d }$=10.7% vs. $\overline{d }$=4.3%). Finally, post-cue digit reports were more frequent than pre-cue digit reports across all combinations of streams × location conditions (all *p*s < 0.04).

### Discussion

Experiment 2 obtained several clear findings. As expected, presenting the selection cue during the interval between two digits, and presenting successive digits at different locations increased the probability of reporting the pre-cue digit. In contrast to Experiment 1, these reports were no longer close to floor level, which made it possible to assess the effects of manipulating the speed of attentional engagement (single versus two RSVPs) for both pre-cue and post-cue digit reports. Critically, the dissociation predicted by the attentional episode account was indeed observed: When the number of streams was increased, the frequency of pre-cue digit reports decreased, whereas the frequency of post-cue digit reports increased. This differential effect was more clearly present on trials where both pre- and post-cue digits were present and appeared at different locations, so that masking was avoided. When they appeared at the same location, pre-cue reports were very infrequent (analogous to Experiment 1).

On trials where RSVP streams contained only the pre-cue or post-cue digit, the probability of reporting the digit was lower in the two-stream condition for both pre-cue and post-cue digits. Importantly, this effect was much more pronounced for pre-cue digits, indicating that the delay of attentional engagement had a stronger effect on the encoding of these items. The fact that post-cue digit reports were also more frequent with a single RSVP stream suggests that positioning the selection cue 50 ms before this digit may have been optimal for its encoding in the single-stream condition. Consequently, the delay of engagement with two streams may have resulted in a slightly weaker activation of the post-cue digit.

## General discussion

The current study was conducted to obtain new insights into the distribution of selective attention in time. More specifically, we investigated the relationship between the speed of attentional engagement and the likelihood to erroneously report distractors instead of the target (distractor intrusions) in RSVP streams. Engagement speed was manipulated by presenting either a single RSVP stream (faster engagement) or two streams (slower engagement). We measured the impact of this manipulation on the probability of reporting distractors that either preceded or followed the target selection cue. In Experiment 1, a pre- or post-target distractor competed with the target for response selection. Here, slower engagement substantially increased post-target intrusions, but had no effect on pre-target intrusions, which were consistently close to floor level. In Experiment 2, the selection cue appeared either after or before objects that could both be reported (pre-cue and post-cue digits). When these two types of digits appeared successively and thus competed with one another, slower engagement increased post-cue reports and decreased pre-cue reports, whereas the reverse pattern was observed with faster engagement. When only the pre-cue or the post-cue digit was present, slower engagement decreased both types of report, but this drop was much steeper for pre-cue reports than for post-cue reports. Thus, across both experiments, delays to attentional engagement increased the proportion of reported objects that appeared after the selection cue relative to reports of objects that appeared prior to the selection cue.

These results are incompatible with the account put forward by Botella et al.^8^, according to which distractor intrusions emerge on trials where attentional selection fails. This account predicts that when the probability of selection failure increases by introducing uncertainty about target location in the two-stream condition, this should generally increase both pre-target and post-target distractor intrusions in Experiment 1, and have similar effects on the probability of pre-cue and post-cue digit reports in Experiment 2. In contrast, our results are entirely compatible with the attentional episode account^9,11^. According to this account, there is no qualitative difference between the processes that result in correct reports and in distractor intrusions. Instead, the probability of reporting a specific object depends on the amount of attentional enhancement that it receives, regardless of its objective status as a target or distractor. Thus, when manipulating the speed of attentional engagement, fast engagement should increase the reporting frequency of pre-target distractors (Experiment 1) and pre-cue digits (Experiment 2), while slow engagement should have the opposite effect, and this is exactly what was observed.

In both experiments, we assumed that increasing the number of streams from one to two slowed attentional engagement^9,12,22,23^, and that the differential effects on pre-target and post-target reports were associated with this delay. However, it is possible that this manipulation may have had additional effects that could also explain our results. For example, spatial attention is already focused on the target’s location when the target appears in the single stream condition, but not in the two streams condition. To demonstrate that it is the speed of engagement, rather than such differences in focused attention was critical in producing our results, we conducted another analysis that assessed the effect of engagement speed in a different way, and independently of spatial attention. Specifically, we relied on the well-established finding that the speed of engagement is linked to probabilistic temporal expectations about the appearance of target objects. In situations where the temporal position of targets among other objects is uncertain, observers are better prepared for later targets, because the absence of earlier targets makes their appearance more likely (a hazard function^24^). Such temporal expectation effects have been shown to affect the speed of engagement as measured with electrophysiological markers^25,26^. We therefore examined whether the temporal position of a target within an RSVP stream has a differential effect on pre-target versus post-target distractor intrusions. The attentional episode account predicts that faster engagement for later as compared to earlier targets should be reflected by fewer post-target intrusions, but should not affect the probability of pre-target intrusions. To test this prediction, we reanalysed the data from Experiment 1, and compared the differences in accuracy and intrusion rates on trials with either pre-target or post-target digit, as function of the targets’ temporal position (early: 5th or 6th position; late: 7th or 8th position). Importantly, this analysis focused solely on blocks with two RSVP streams, to keep the distribution of spatial attention constant, and to ensure that the target (and pre-target distractor) did not benefit from focused spatial attention.

**Figure 7:**
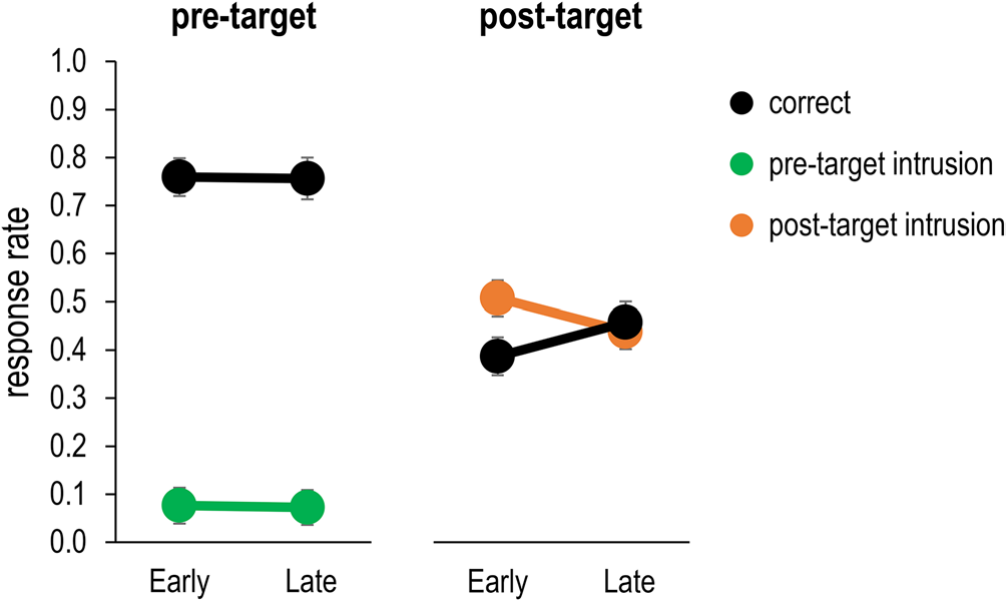
Frequency of correct responses (black lines), pre-target intrusions (green lines) and post-target intrusions (orange lines) in Experiment 1, as a function of the distractor condition (pre-target digit vs. post-target digit) and the target’s temporal position in the RSVP stream (early vs. late, i.e., 5th and 6th positions vs. 7th and 8th positions). Error bars denote one standard error.

The results of these comparisons (Fig. 7) were fully in line with the attentional episode account. For trials with a post-target digit, early targets were associated with lower accuracy (*M* = 38.7% vs. *M* = 45.7%) and higher intrusion rates (*M* = 50.8% vs. *M* = 43.8%). In contrast, in trials with a pre-target digit, there were no differences between early and late targets in accuracy (*M* = 75.9% vs. *M* = 75.6%) or intrusion rates (*M* = 7.6% vs. *M* = 7.2%). A statistical analysis confirmed this pattern. A two-way ANOVA revealed that the interaction between distractor type and temporal position was significant for both accuracy and intrusion rates, *F*(1,31) = 10.07, *p* = 0.003, ${\eta }_{p}^{2}$=0.25, and *F*(1,31) = 9.41, *p* = 0.004, ${\eta }_{p}^{2}$=0.23, respectively. Follow-up analysis indicated that the simple effects were significant for trials with post-target distractors, both *p*s < 0.001, but not for trials with pre-target distractors, both *F*s < 1. The results supported the same conclusions when only very early and very late targets were compared (5th versus 8th position).

By providing support for the attentional episode account, the current results have important general implications for attention research. Many models of attention view selection as a discrete stage that gates the access of sensory inputs to capacity-limited perceptual processing (see^27^ for review). The current study provides further support for the alternative view that attentional selectivity is not temporally discrete, but unfolds in real time during a critical period (attentional episode). Another conclusion from the current study is that distractor intrusions can be used to investigate the exact time course of these episodes, and their temporal variability. Given the close association between the frequency of different types of reports and the speed of attentional engagement demonstrated here, the pattern of distractor intrusions can be used to determine whether a certain manipulation affects the temporal dynamics of attentional processes. Distractor intrusion paradigms should become important parts of the attention researcher’s toolbox when investigating the time course of attentional selectivity, alongside other temporally continuous electrophysiological markers such as the N2pc component. However, unlike the N2pc, distractor intrusions paradigms are cheaper and more flexible to use, as they do not require EEG recording equipment and are not limited to lateralized displays.

Distractor intrusions can therefore facilitate research into the temporal dynamics of attention, which in turn, can help resolve longstanding debates in the attention literature, such as the mechanisms responsible for the attentional blink (AB). In a standard AB paradigm, detection of the second of two targets is strongly impaired when it appears between 200 and 500 ms after the first target. It is still unclear which processes are disrupted during the blink period (see^28^ for a recent review). AB studies that employed distractor intrusions in RSVP streams^29,30^ can provide important clues towards a definitive answer. These studies showed that inaccurate target reports during the blink period are linked to a higher percentage of post-target intrusions. Based on the current results, such a pattern would suggest that attentional engagement is delayed during the AB, which has indeed been suggested^17,28,31^.

In addition to providing support for a strong link between distractor intrusions and attentional episodes, the current results also suggest that some previous ideas developed within this framework may need to be revised. Specifically, the low frequency of pre-target intrusions has previously been explained by assuming that representations of pre-target distractors are fragile and thus prone to strong backward masking by subsequent items^9,12^. While Experiment 2 revealed that masking does indeed reduce these intrusions, it also suggested that the representations of masked pre-cue digits can persist for longer duration than previously thought. When a pre-cue digit was followed by a letter at the same location and should therefore have been strongly masked, it was still reported on approximately 60% of all trials, indicating that masking did not prevent its encoding in working memory on these trials. This suggests that in standard distractor intrusion paradigms where pre-target distractors are followed by targets, these distractors are also frequently encoded. Thus, the low frequency of pre-target intrusions may not be caused by backward masking preventing their access to working memory, but rather by competitive interactions between distractor and target representations within working memory.

In summary, the current results support the notion that attentional episodes and their temporal variability play a major role in the conscious perception of visual objects in RSVP streams, and in producing distractor intrusions. It should be noted that the pattern of distractor intrusion effects observed in the present study may be specific to the particular type of stimuli used (alphanumerical characters). For example, it has previously been suggested that pre-target distractor intrusions may be much more frequent than post-target intrusions when more complex stimuli such as real-world objects and scenes are employed^32^. It will be important to determine in future studies whether and how this is linked to the speed of attentional engagement. More generally, the current study illustrates that the pattern of distractor intrusion effects can index the speed of attentional engagement, and thus provide a new and useful tool for research into the temporal dynamics of selective attention.

## Methods

### Ethics

All methods used in the two experiments reported here were approved by the Psychological Sciences Departmental Ethics Committee at Birkbeck, University of London. The experiment was conducted in line with the ethical guidelines laid down in the 6th Declaration of Helsinki. All participants provided voluntary and informed consent to participate before taking part in the experiments.

### Experiment 1

#### Sample size selection

We conducted a power analysis based on the results obtained in Experiment 1 of Ludwici and Holcombe, and specifically, on the observed difference in the temporal position of the selected object (latency) as function of increased spatial uncertainty about the target’s location. The effect size for this result was dz = 1.87. A power analysis using G*power^33^ and an effect half of this size suggested a minimum sample size of N = 11 is required to achieve 80% power. However, due differences in method, we recruited a much larger sample size of N = 32, which can be used to detect much smaller effects.

#### Participants

Participants were 32 (13 women) volunteers (Mage = 30.6, SD = 9.4) who participated for £5 or course credits. All reported normal or corrected-to-normal visual acuity.

#### Apparatus

Stimuli were presented on a 24-inch BenQ LED monitor (120 Hz; 1920 × 1080 screen resolution) attached to a SilverStone PC in a dimly lit room, with participant viewing distance at approximately 80 cm. Manual responses were registered via a standard computer keyboard.

#### Stimuli and design

All methods used in this experiment, and the subsequent experiment, were approved by the institution’s departmental ethical guidelines committee at Birkbeck, University of London. Participants had to report as accurately as possible the numerical value of a digit (report feature) that appeared inside a square cue (selection feature), by pressing the corresponding keyboard button. Participants’ left and right hands rested on the top row number keypads in the keyboard (using their ring finger, middle finger, and index finger) such that they didn’t have to look at the keyboard when responding. These targets were presented unpredictably in a single RSVP stream that appeared in the center of the screen or two RSVP streams on the left and right to fixation. Manual responses were executed without time pressure at the end of each trial. The sequence of events is illustrated in Fig. 3. Each trial began with the presentation of a fixation display (a grey 0.2° × 0.2° “ + ” sign at the center of the screen). Then, after 500 ms, 8 to 11 RSVP frames appeared. Each frame appeared for 50 ms, followed by an ISI of 50 ms. The response screen was identical to the fixation display and remained present until a response was registered. Following this response, a blank screen appeared for 800 ms before a new trial started.

The experiment included 10 practice trials followed by 400 experimental trials, divided into 50-trial blocks. For half the participants, the target was embedded among a single RSVP stream for the first four blocks and among two RSVP streams for the rest. For the other half, this order was reversed. Instructions about this change were given before the beginning of the 5th block, followed by additional 5 practice trials. Participants were allowed to take self-paced breaks between blocks. Preliminary analysis indicated that the order of the blocks (single stream first or two streams first) did not affect any of the results in this or the following experiment. Therefore, all analyses were collapsed across this variable.

All stimuli in the RSVP streams were grey (CIE colour coordinates: 0.309/0.332, luminance 46.6 cd/m^2^). On trials with a single RSVP stream, the RSVP consisted of a single alphanumeric character (1.3° in height) per frame, appearing in the center of the screen. Letters in each stream were randomly selected without replacement from a 24-letter set (all English alphabet letters, excluding I and O). Digits were selected without replacement from a set of 9 digits (1–9), except for the target digit which was restricted to a set of 6 digits (2, 3, 4, 6, 7, and 8). The target digit appeared with equal probability and unpredictably in the 5th, 6th, 7th, or 8th frame within the RSVP stream. This target frame contained a single digit that appeared within a square outline (1.5° in side, 4 pixel line-width). Each target digit was preceded by one to three digits which appeared in random temporal positions in the RSVP stream. These digit distractors were included to ensure that allocation processes would be guided by the selection feature (square), rather than by alphanumerical category (i.e., attending to the first digit in the stream). On 20% of the trials, the category of the frame immediately preceding (pre-target) and immediately following (post-target) the target were letters, thereby preventing pre-target intrusions and post-target intrusions (baseline condition). On 40% of the trials the pre-target was a digit, and the post-target was a letter (pre-target condition), and on the rest of the trials (40%) the pre-target was a letter, and the post-target was a digit (post-target condition). The last two frames contained only letters.

Trials where the target was embedded among two stream RSVP trials were identical to single RSVP trials except for the following differences. Each frame consisted of two alphanumeric characters appearing at a center-to-center distance of 4.5° to the left and right of fixation. Letter selection was restricted such that the same letter could not appear in both streams at the same time. The target frame contained one digit (within a square) in one stream and one letter in the other stream. On baseline trials, the pre-target and post-target frames included only letters. In the digit pre-target and digit post-target conditions only the character that shared the target’s location was a digit whereas the other was a letter. The last two frames always included two letters.

#### Statistical analysis

In Experiment 1, we first examined whether the presence of a pre-target and post-target distractor reduced accuracy. To do so, we conducted a series of dependent sample t-tests to compare accuracy when a distractor was present versus when it was absent (the baseline condition). The comparison was done relative to the respective baseline condition, for example, the single-stream pre-target condition was compared to the single-stream baseline condition. The combination of distractor condition (pre-target, post-target) and the number of streams (single-stream, two-streams) yielded four comparisons in total.

Second, we excluded baseline accuracy data, and entered accuracy as a dependent variable to an ANOVA with distractor condition (pre-target, post-target) and number of streams (single vs. two) as within-subject factors. Thirdly, we entered intrusion rates on the distractor present trials to an ANOVA with the same variables. For these ANOVA models, significant interactions were followed by a simple-effects analysis.

### Experiment 2

#### Sample size selection

The sample size in Experiment 2 was selected based on the effect size of the stream × distractor interaction (${\eta }_{p}^{2}$=0.45) observed in Experiment 1. A power analysis indicated that a sample of 16 participants would provide 90% power to find a significant effect with similar effect size.

#### Participants

Participants were 16 (9 women) volunteers (Mage = 31.6, SD = 7.3) who participated for £8 or course credits. All reported normal or corrected-to-normal visual acuity.

#### Apparatus, stimuli and design

The apparatus, stimuli, and design were identical to Experiment 1 except for the following differences (see Fig. 5). In the single-stream RSVP condition, the stimuli randomly appeared either 0.55° to the left or right of the centre of the screen, all within the area covered by the selection cue. In the two-streams condition, the same distance was calculated from the centre of the selection cue (4.5° to the left and right of fixation), with stimuli appearing either 5.05° or 3.95° to the left or right of fixation.

The experiment included 10 practice trials followed by 640 experimental trials, divided into 80-trial blocks. The target was once again defined by a surrounding square. However, this target-defining square was orange (CIE colour coordinates: 0.568/0.401) for half of the participants and green (0.306/0.615) for the rest. The two colours were approximately equiluminant (~ 47 cd/m^2^). This change was introduced in preparation for a future EEG experiment where colour equiluminance is critical when measuring lateralized event-related potentials. In the single RSVP stream condition, the target frame contained only the square in the target-defining colour. In the two RSVP streams condition, the target frame contained two squares, one orange and one green. Preliminary analysis indicated that the colour of the selection cue (orange or green) had no effect on pre-cue or post-cue report rates. Therefore, we collapsed all the data across this between-subject factor.

Unlike Experiment 1, the selection cue never coincided with any digit or letter in the RSVP streams, but was presented during the 50 ms period between two successive items. On 80% of trials, the selection cue appeared during the interval between two digits (pre-cue and post-cue digits). On the rest of the trials, the selection cue either appeared after a digit and before a letter (pre-cue digit only condition), or after a letter and before a digit (post-cue digit only condition). Although there was no objectively correct response as the cue did not coincide with any digit, participants were not informed of this fact. Instead, they were instructed (as in Experiment 1) to report which digit they saw inside within the relevant coloured square by pressing the corresponding keyboard button.

#### Statistical analysis

In Experiment 2 we separate analyses on trials where both the pre-cue and post-cue items were digits and on trials where either the post-cue or the pre-cue items were digits. For each dataset, we conducted a three-way ANOVA with reported digit (post-cues vs. pre-cues), number of streams (single-stream vs. two-streams) and pre-cue/post-cue location relative to one another (same vs. different) as within-subject independent variables. A Significant three-way interaction was followed-up using separate two-way ANOVAs, one for each type of reported digit, as function of the number of streams and pre-cue/post-cue locations. A significant two-way interaction was followed-up with simple-effects analysis. Finally, for sake of completeness, we examined whether post-cue reports exceeded pre-cue reports in all streams-number × pre-cue/post-cue location conditions.

## Data Availability

The data from both experiments is available at https://doi.org/10.6084/m9.figshare.21277731.
